# Development and clinical validation of a nursing risk prediction model for chemotherapy-induced febrile neutropenia in patients with cancer

**DOI:** 10.3389/fcell.2026.1779406

**Published:** 2026-04-22

**Authors:** Fang Xie, Dongmei Zheng, Na Chen, Jie Zhang, Nan Wang, Lihuai Wang, Qin Yi

**Affiliations:** 1 Cancer Center, The First Hospital of Hunan University of Chinese Medicine, Changsha, Hunan, China; 2 Hematological Oncology Department, The First Hospital of Hunan University of Chinese Medicine, Changsha, Hunan, China; 3 Hunan University of Chinese Medicine, Changsha, Hunan, China

**Keywords:** chemotherapy, clinical prediction model, febrile neutropenia, oncology nursing, risk stratification

## Abstract

**Background:**

Febrile neutropenia (FN) is one of the most serious yet potentially preventable complications of systemic chemotherapy. However, practical tools that support nursing-led early risk stratification and workflow-ready preventive actions remain limited.

**Methods:**

We performed a real-world cohort study including 2,125 patients with cancer receiving systemic chemotherapy at a single institution. Patients were randomly split (7:3) into a derivation cohort (70%) and an internal validation cohort (30%). Candidate predictors were prespecified to ensure bedside nursing assessability and routine clinical availability, incorporating both conventional clinical/laboratory factors and nursing-relevant indicators (e.g., nutritional risk, mucositis, and self-monitoring adherence). Predictors were selected using penalized regression, followed by multivariable logistic modeling to estimate FN risk in the first evaluable chemotherapy cycle. Model performance was assessed by discrimination and calibration, and clinical utility was examined using decision curve analysis. A nomogram and risk-stratified nursing pathways were developed to translate predicted risk into actionable surveillance and preventive care.

**Results:**

The final nursing-oriented model showed good discrimination and satisfactory calibration in both the derivation and validation cohorts. Decision curve analysis indicated net benefit across a clinically relevant range of threshold probabilities. Risk stratification based on predicted probabilities was associated with graded increases in FN incidence and adverse clinical outcomes.

**Conclusion:**

This nursing-oriented FN prediction model provides individualized early-cycle FN risk estimation and operational risk stratification to support targeted surveillance and preventive nursing interventions. External validation across diverse institutions and nursing documentation systems is warranted.

## 1 Introduction

Systemic chemotherapy remains a cornerstone of treatment for both solid tumors and hematologic malignancies (Sheehy et al., 2025), yet its effectiveness is frequently constrained by treatment-related myelotoxicity (Sugaya et al., 2025). Among these toxicities, febrile neutropenia (FN) is one of the most serious and, at least in part, preventable complications. FN often triggers urgent assessment, hospital admission, empirical broad-spectrum antimicrobial therapy, and intensive monitoring and supportive care. It can also lead to chemotherapy delays or dose reductions, compromising relative dose intensity and potentially undermining long-term oncologic control. From both clinical and nursing perspectives, FN is rarely an isolated episode; it can initiate a cascade that includes infection progression, sepsis, treatment interruption, and sustained deterioration in quality of life across the course of care (Gao et al., 2025; Gallardo-Pizarro et al., 2025).

Current FN management relies on risk stratification, administration of prophylactic granulocyte colony-stimulating factor (G-CSF) for selected patients, standardized infection work-up, and rapid response protocols designed to reduce severe infections while preserving antitumor efficacy (Sandherr et al., 2025). However, early identification of patients at high risk before FN occurs and the rational allocation of limited nursing resources during chemotherapy remain challenging (Klastersky et al., 2016). FN risk is multifactorial, reflecting baseline immune reserve, comorbidity burden, nutritional status, tumor type, chemotherapy intensity, and cycle-related marrow suppression, as well as mucosal barrier integrity and exposure-related infection risks. In practice, delays in symptom recognition and care seeking, which are often linked to atypical fever presentation, limited self-monitoring capacity, or suboptimal adherence to monitoring plans, may accelerate infection progression and worsen outcomes. For oncology nurses, the practical question is how to integrate routine clinical data with bedside nursing assessments into actionable early-warning signals that guide follow-up frequency, temperature monitoring, education priorities, and timely escalation for medical evaluation (Morimoto et al., 2024).

Existing tools only partially address this need. Commonly used clinical scores and decision aids are primarily designed to stratify complications after FN has occurred or to support therapeutic decision-making, rather than to estimate FN risk prospectively during chemotherapy cycles (Jia et al., 2024; Thungthong et al., 2023). Although several prediction models have been proposed, translation into routine practice is often limited by restricted variable availability, heterogeneous populations, inconsistent endpoint definitions, and variable performance across settings. Importantly, many models rely predominantly on pharmacologic and laboratory predictors and incorporate few variables that are nurse-assessable, nurse-interpretable, or nurse-modifiable (Zatarah et al., 2022). Nursing-relevant domains, such as nutritional risk, oral mucositis and barrier injury, catheter-related care risks, symptom burden, self-monitoring capacity, and adherence, are closely linked to infection vulnerability and delays in care seeking. However, they are frequently underrepresented in existing models (Chetry et al., 2025). As a result, statistically significant predictors do not consistently translate into clear nursing workflows or intervention pathways and may fail to capture the distinctive contribution of oncology nursing to FN prevention (Zhu et al., 2022).

To address these gaps, this study aimed to develop and internally validate a nursing-oriented FN risk prediction model that is interpretable, feasible within routine nursing workflows, and explicitly designed for early-cycle risk assessment (Venäläinen et al., 2022). We combined conventional clinical factors (e.g., regimen-related myelotoxicity risk, comorbidity profile, and key baseline and on-treatment laboratory indicators) with structured nursing assessment variables, including nutritional status, mucosal barrier injury, catheter-related risk, symptom clusters, and self-management ability, to form a multidimensional candidate predictor set. After standardized data cleaning and prespecified missing-data handling, penalized regression and multivariable modeling were used to derive a final model for individualized FN risk estimation in the first evaluable chemotherapy cycle (Hsu et al., 2025). Model discrimination, calibration, and clinical utility were assessed in an internal validation cohort, and a risk-stratification scheme was constructed to support corresponding nursing management pathways.

## 2 Methods

### 2.1 Study design and setting

This retrospective, real-world, single-center cohort study developed and internally validated a nursing-oriented prediction model for chemotherapy-related febrile neutropenia (FN). Data were extracted from the electronic medical record (EMR), the laboratory information system (LIS), and the nursing information system of the oncology department in a tertiary hospital. Linking these sources enabled consistent capture of treatment exposure, laboratory indicators, and structured nursing assessments within the same prespecified observation window for each patient and chemotherapy cycle (Tahir et al., 2025).

The analytic unit was the patient. For patients receiving multiple chemotherapy cycles during the study period, only the first evaluable cycle within the observation window was retained for model development to avoid non-independence from repeated cycles and to ensure uniform predictor ascertainment preceded outcome assessment. Accordingly, the model is intended for early-cycle risk stratification to support initial preventive nursing planning and resource allocation (Chen et al., 2025).

All eligible patients meeting prespecified criteria during the study period were included (N = 2,125). The cohort was randomly split (7:3) into a derivation cohort for model development and an internal validation cohort for model evaluation. Randomization used a computer-generated sequence, stratified by tumor category (solid vs. hematologic malignancies) and chemotherapy regimen myelotoxicity risk level (high, intermediate, and low) to balance key prognostic factors across cohorts. Baseline demographic, clinical, and nursing-related characteristics were compared between cohorts to assess balance; no clinically meaningful imbalances were observed.

**FIGURE 1:**
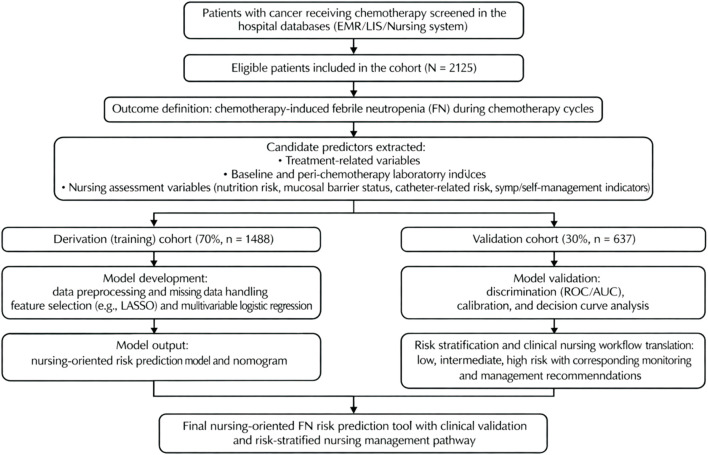
Study flowchart.

Because this was a retrospective cohort study including all consecutive eligible patients within a predefined time frame (1 January 2021 to 31 December 2024), no formal *a priori* sample-size calculation was performed. To reduce overfitting, model complexity was constrained by the observed number of FN events (E = 214) and recommendations for penalized regression and multivariable logistic modeling (Tahir et al., 2025). The overall workflow, from cohort assembly to model development, internal validation, and translation to risk-stratified nursing pathways, is shown in Figure 1.

### 2.2 Participants and eligibility criteria

Adult patients with cancer who received systemic cytotoxic chemotherapy in the participating oncology department were eligible (Orbaugh et al., 2024). Diagnoses, chemotherapy regimens, laboratory tests, and nursing assessment data were obtained from hospital information systems and linked at the patient level using unique identifiers.

Inclusion criteria were (1) pathologically or clinically confirmed malignancy; (2) age ≥18 years; (3) receipt of intravenous or oral cytotoxic chemotherapy with clearly documented regimen type, administration date, and cycle structure; (4) completion of at least one evaluable chemotherapy cycle with available body-temperature records and complete blood counts (including absolute neutrophil count [ANC]) within the follow-up window; and (5) availability of core nursing assessment indicators relevant to FN prevention (e.g., nutritional risk, mucosal barrier status, catheter-related risk, and self-management capacity).

Exclusion criteria were applied to reduce outcome misclassification and major sources of non-chemotherapy-related fever: (1) active infection requiring ongoing intravenous anti-infective therapy before chemotherapy initiation; (2) persistent baseline fever or profound immunodeficiency at enrollment precluding attribution of subsequent events to chemotherapy-induced myelosuppression; (3) hematopoietic stem-cell transplantation or CAR-T therapy due to distinct immune status and care pathways; (4) cycles with fever clearly attributable to vaccination, major surgery/trauma, or immediate postoperative causes based on physician and nursing documentation; (5) pregnancy or lactation; (6) severe cognitive or psychiatric disorders precluding reliable nursing assessment; and (7) extensive missing data on key predictors or outcomes that could not be addressed using prespecified procedures. For patients receiving multiple cycles, only the first evaluable cycle was retained for analysis, as described above.

### 2.3 Outcome definition and follow-up window

The primary outcome was chemotherapy-related FN occurring within the prespecified observation window for the evaluable cycle. FN was defined as fever plus neutropenia within a clinically plausible temporal window. Fever was defined as a single oral temperature ≥38.3 °C or a sustained temperature ≥38.0 °C for ≥1 h, based on routine inpatient or outpatient temperature records. Neutropenia was defined as ANC <0.5 × 10^9^/L, or ANC 0.5–1.0 × 10^9^/L with an anticipated decline to <0.5 × 10^9^/L within 48 h inferred from serial blood counts and clinician notes.

To minimize misclassification, the temporal relationship between fever and neutropenia was cross-checked using time-stamped temperature and laboratory records. Episodes documented as perioperative fever, vaccine-related fever, or other non-chemotherapy causes were excluded based on medical and nursing records. The first time point at which the FN definition was met within the cycle was recorded as the event time.

Follow-up was defined at the cycle level. For each patient, follow-up began on the day of chemotherapy administration for the evaluable cycle. It ended at the last planned day of that cycle or the start of the next cycle, whichever occurred first. Patients meeting the FN definition within this window were classified as events; those not meeting the definition by the end of follow-up were classified as non-events.

### 2.4 Candidate predictors: nursing-oriented framework

**FIGURE 2:**
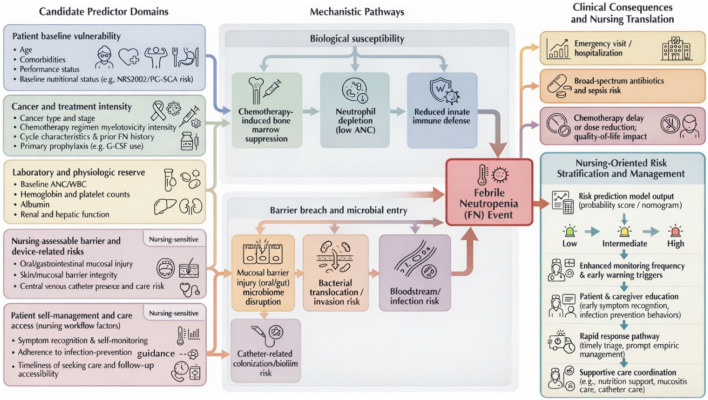
Nursing-oriented framework.

Candidate predictors were prespecified based on established FN pathogenesis and a pragmatic principle: the model should prioritize variables that are routinely available and actionable in nursing workflows (assessable, interpretable, and, where possible, modifiable through nursing practice). The framework conceptualizes two primary pathways leading to FN: (1) bone marrow suppression and reduced immune reserve and (2) mucosal barrier disruption with subsequent microbial invasion. Nursing workflow-related factors were explicitly integrated to enable model outputs to be translated into stratified nursing actions rather than serving solely as predictions. Figure 2 summarizes this nursing-oriented framework and its linkage to FN events and downstream clinical consequences.

Candidate variables were organized into five domains based on this framework. The first domain captured baseline susceptibility, including age, comorbidity burden, functional status, and pretreatment nutritional status, reflecting host tolerance to myelosuppression and infection stress. The second domain reflected tumor- and treatment-related exposure, including tumor type and stage, regimen myelotoxicity risk classification, cycle characteristics, prior FN history, and prophylactic G-CSF use. The third domain summarized routinely measured laboratory and physiological reserve indicators available at baseline or early in the cycle (e.g., ANC, white blood cell count, hemoglobin, platelet count, albumin, and basic liver/kidney function), reflecting marrow reserve, nutritional/metabolic status, and drug clearance capacity.

The fourth domain captured barrier- and device-related risks directly observable in nursing care, including the severity of oral/gastrointestinal mucositis or barrier injury and the presence of central venous catheters with care-related risk profiles. The fifth domain covered self-management and access to care, including symptom recognition/reporting, adherence to infection-prevention behaviors, home temperature monitoring, and patterns of follow-up and healthcare access, which represent intervention points for nursing education and follow-up once risk stratification is available.

Predictor ascertainment was anchored to the start of the evaluable cycle. Baseline laboratory values were defined as the most recent result within 7 days prior to cycle day 1; if multiple results were available, the value closest to day 1. Structured nursing assessments were defined as the standardized pretreatment nursing assessment completed within 24 h before chemotherapy initiation; if unavailable, the closest within 48 h. Standardized operational definitions and coding rules were applied to reduce information bias. For nursing-modifiable variables, clinically meaningful categories were retained to preserve interpretability and facilitate direct mapping from predicted risk to nursing management pathways.

### 2.5 Data processing and missing data handling

After extraction, data underwent consistency and plausibility checks, including unit harmonization, range checks to identify implausible values, time-stamp verification, and removal of duplicate or clearly erroneous entries. Continuous variables were examined for distributional characteristics and transformed or standardized when necessary for model fitting, while preserving clinically interpretable scales where possible. Categorical variables were coded according to prespecified clinical stratification rules to ensure reproducibility.

Missing data were handled using a prespecified strategy. We quantified missingness for each variable and assessed missing-data patterns. Under an assumption of missing at random conditional on observed covariates, variables with acceptable missingness were imputed using multiple imputation. Imputation used multivariable imputation by chained equations (MICE) with predictive mean matching for continuous variables and logistic/multinomial models for categorical variables, with m = 20 imputations. It included variables related to both outcome and missingness (including the outcome indicator) to reduce bias. Variables with missingness exceeding 30% were excluded or consolidated following clinical and nursing review. To avoid information leakage, the imputation model was fit in the derivation cohort and applied to the validation cohort using identical settings. Sensitivity analyses compared the primary approach with complete-case analysis and single imputation using median/mode.

### 2.6 Model development and feature selection

Model development was performed in the derivation cohort to achieve stable prediction and bedside interpretability. Candidate predictors were encoded according to measurement type. Continuous predictors were generally retained as continuous and not dichotomized arbitrarily.

Feature selection and estimation were conducted in two steps. First, penalized logistic regression using the least absolute shrinkage and selection operator (LASSO) was used to reduce dimensionality, address multicollinearity, and limit overfitting. Prior to penalization, continuous predictors were z-score standardized. The penalty parameter (λ) was selected via 10-fold cross-validation using the 1-SE rule. Second, multivariable logistic regression was fitted using the selected predictors to estimate each patient’s probability of FN in the evaluable cycle. Final model selection balanced statistical performance with clinical feasibility and nursing relevance, excluding predictors that were impractical to obtain or lacked clear nursing implications.

To quantify the incremental contribution of nursing-assessable information, we developed (i) a baseline model including treatment-related and routine laboratory predictors and (ii) a nursing-enhanced model that additionally incorporated structured nursing assessment variables. The nursing-enhanced model was prespecified as the primary model for presentation. Regression coefficients were used to derive a nomogram and a bedside-friendly scoring scheme.

Internal validation in the derivation cohort used bootstrap resampling with 1,000 repetitions to estimate optimism-corrected discrimination and calibration. When indicated, coefficient shrinkage was applied using uniform shrinkage based on the bootstrap-derived calibration slope to reduce overfitting.

### 2.7 Internal validation and clinical utility

After model development in the derivation cohort, performance was evaluated in the held-out validation cohort created by the prespecified 7:3 split. Discrimination was quantified using the area under the receiver operating characteristic curve (AUC). Calibration was assessed by comparing predicted versus observed FN risk across deciles of predicted probability and by inspecting calibration plots, reporting the calibration intercept and slope.

Clinical utility was evaluated using decision curve analysis (DCA), which estimates net benefit across a range of threshold probabilities representing clinically meaningful trade-offs between unnecessary intensified management and missed high-risk patients. Net benefit curves were compared with default strategies of managing all patients as high risk versus managing none.

To translate predicted probabilities into bedside decision support, we constructed a three-tier risk-stratification scheme (low, intermediate, and high). Thresholds were selected using a transparent process integrating (i) the range of threshold probabilities where DCA demonstrated meaningful net benefit, (ii) the distribution of predicted risks to ensure separation of observed FN incidence across strata, and (iii) feasibility considerations for differentiating nursing actions across strata. The resulting strata were evaluated for graded differences in FN incidence and clinically relevant adverse outcomes.

Finally, we mapped each risk stratum to prespecified nursing management pathways to support implementation from prediction to action. Low-risk patients were managed with routine monitoring and standard infection-prevention education. Intermediate-risk patients were assigned enhanced follow-up intensity with reinforced temperature self-monitoring, nutrition-related support, and self-management coaching. High-risk patients were prioritized for intensified surveillance with early escalation triggers and closer post-discharge follow-up coordinated with the oncology care team according to local protocols. This framework was designed to enable consistent operationalization of risk prediction into nursing workflows rather than producing risk estimates alone.

## 3 Results

### 3.1 Cohort construction and baseline characteristics

A total of 2,125 chemotherapy-treated adult patients with cancer met the eligibility criteria and were included in the analysis. Following the prespecified 7:3 split, 1,488 patients comprised the derivation cohort, and 637 comprised the validation cohort. Baseline demographic, disease-related, treatment-related, laboratory, and nursing assessment profiles were well balanced between cohorts, consistent with the stratified random allocation procedure and supporting stable model development and internal validation (Table 1).

**TABLE 1:** Baseline patient characteristics and chemotherapy-related profiles in the overall cohort and by derivation and validation cohorts.

Characteristic	Overall (N = 2,125)	Derivation cohort (n = 1,488)	Validation cohort (n = 637)
Age, years, mean ± SD	57.2 ± 12.1	57.1 ± 12.0	57.5 ± 12.3
Female sex, n (%)	1,017 (47.9)	709 (47.6)	308 (48.4)
Body mass index, kg/m^2^, mean ± SD	23.6 ± 3.4	23.5 ± 3.4	23.8 ± 3.5
ECOG performance status 0–1, n (%)	1,764 (83.0)	1,236 (83.1)	528 (82.9)
ECOG performance status ≥2, n (%)	361 (17.0)	252 (16.9)	109 (17.1)
Current or former smoker, n (%)	623 (29.3)	436 (29.3)	187 (29.4)
Cardiovascular disease, n (%)	368 (17.3)	258 (17.3)	110 (17.3)
Diabetes mellitus, n (%)	312 (14.7)	219 (14.7)	93 (14.6)
Chronic kidney disease, n (%)	128 (6.0)	90 (6.0)	38 (6.0)
Chronic lung disease (COPD/asthma), n (%)	146 (6.9)	102 (6.9)	44 (6.9)
Cancer type, n (%)			
Lung	412 (19.4)	289 (19.4)	123 (19.3)
Breast	365 (17.2)	256 (17.2)	109 (17.1)
Colorectal	332 (15.6)	232 (15.6)	100 (15.7)
Gastric	256 (12.0)	179 (12.0)	77 (12.1)
Hematologic	286 (13.5)	200 (13.4)	86 (13.5)
Gynecologic	214 (10.1)	150 (10.1)	64 (10.0)
Other	260 (12.2)	182 (12.2)	78 (12.2)
Cancer stage, n (%)			
Localized	514 (24.2)	360 (24.2)	154 (24.2)
Locally advanced	721 (33.9)	505 (33.9)	216 (33.9)
Metastatic	890 (41.9)	623 (41.9)	267 (41.9)
Line of systemic therapy, n (%)			
First line	1,422 (66.9)	996 (66.9)	426 (66.9)
Second line	503 (23.7)	352 (23.7)	151 (23.7)
Third line or later	200 (9.4)	140 (9.4)	60 (9.4)
Chemotherapy myelotoxicity risk, n (%)			
High	468 (22.0)	328 (22.0)	140 (22.0)
Intermediate	1,064 (50.1)	745 (50.1)	319 (50.1)
Low	593 (27.9)	415 (27.9)	178 (27.9)
Cycle at enrollment, n (%)			
Cycle 1	702 (33.0)	492 (33.1)	210 (33.0)
Cycles 2–3	884 (41.6)	619 (41.6)	265 (41.6)
Cycle ≥4	539 (25.4)	377 (25.3)	162 (25.4)
Prior febrile neutropenia history, n (%)	248 (11.7)	174 (11.7)	74 (11.6)
Primary prophylactic G-CSF use, n (%)	498 (23.4)	349 (23.5)	149 (23.4)
Antibacterial prophylaxis, n (%)	179 (8.4)	125 (8.4)	54 (8.5)
Prior radiotherapy within 6 months, n (%)	517 (24.3)	362 (24.3)	155 (24.3)
Central venous catheter, n (%)	812 (38.2)	569 (38.2)	243 (38.1)
Mucositis grade ≥2 during cycle, n (%)	317 (14.9)	222 (14.9)	95 (14.9)
Nutrition risk (NRS 2002 ≥ 3 or PG-SGA B/C), n (%)	604 (28.4)	423 (28.4)	181 (28.4)
Limited self-monitoring adherence*, n (%)	356 (16.8)	249 (16.7)	107 (16.8)
Inpatient chemotherapy administration, n (%)	784 (36.9)	549 (36.9)	235 (36.9)
Planned relative dose intensity ≥85%, n (%)	1,612 (75.9)	1,129 (75.9)	483 (75.8)
Use of corticosteroids (≥10 mg prednisone-equivalent) during cycle, n (%)	267 (12.6)	185 (12.4)	82 (12.9)
Baseline absolute lymphocyte count, ×10^9^/L, median (IQR)	1.41 (1.08–1.82)	1.40 (1.08–1.80)	1.43 (1.09–1.85)
Neutrophil-to-lymphocyte ratio, median (IQR)	2.38 (1.65–3.42)	2.37 (1.64–3.41)	2.41 (1.67–3.46)
eGFR, ml/min/1.73 m^2^, mean ± SD	92.8 ± 18.6	93.1 ± 18.4	92.1 ± 19.1
Total bilirubin, µmol/L, median (IQR)	10.7 (7.9–14.8)	10.6 (7.9–14.6)	10.9 (8.0–15.2)
Baseline temperature, °C, mean ± SD	36.71 ± 0.32	36.70 ± 0.32	36.73 ± 0.31
Oral care education documented before cycle, n (%)	1,698 (79.9)	1,191 (80.0)	507 (79.6)
24/7 hotline access documented, n (%)	1,479 (69.6)	1,036 (69.6)	443 (69.5)
Baseline ANC, ×10^9^/L, median (IQR)	3.24 (2.61–4.05)	3.22 (2.60–4.02)	3.29 (2.66–4.10)
Baseline WBC, ×10^9^/L, mean ± SD	5.88 ± 1.76	5.86 ± 1.74	5.94 ± 1.81
Hemoglobin, g/L, mean ± SD	118.6 ± 17.9	118.4 ± 17.7	119.1 ± 18.3
Platelets, ×10^9^/L, median (IQR)	223 (176–287)	222 (175–285)	225 (178–291)
Albumin, g/L, mean ± SD	39.2 ± 4.8	39.1 ± 4.8	39.4 ± 4.9
Creatinine, µmol/L, median (IQR)	74 (63–88)	74 (63–87)	75 (64–90)
ALT, U/L, median (IQR)	22 (16–31)	22 (16–31)	23 (16–32)
C-reactive protein, mg/L, median (IQR)	4.6 (2.1–9.3)	4.5 (2.1–9.2)	4.8 (2.2–9.6)

“*” denotes limited self-monitoring adherence was defined as documented suboptimal adherence to temperature monitoring and/or delayed reporting of infection-related symptoms during the chemotherapy cycle.

Overall, the cohort was composed predominantly of middle-aged and older adults (mean age, 57.2 ± 12.1 years), with a near-even sex distribution (female, 47.9%). Most patients had Eastern Cooperative Oncology Group (ECOG) performance status 0–1 (83.0%) and were receiving first-line systemic therapy (66.9%). Tumor types covered a broad spectrum, including lung (19.4%), breast (17.2%), colorectal (15.6%), gastric (12.0%), hematologic malignancies (13.5%), and gynecologic tumors (10.1%), with stages spanning localized (24.2%), locally advanced (33.9%), and metastatic disease (41.9%). Regimens were distributed across myelotoxicity risk levels (high, 22.0%; intermediate, 50.1%; low, 27.9%), providing variability in exposure intensity for subsequent risk modeling.

Nursing-relevant characteristics showed clinically meaningful prevalence and variability, including central venous catheter use (38.2%), mucositis grade ≥2 (14.9%), nutritional risk (28.4%), and limited self-monitoring adherence (16.8%). Key baseline laboratory indicators [e.g., ANC, WBC, hemoglobin, platelets, albumin, renal and hepatic indices, and C-reactive protein (CRP)] were generally within clinically plausible ranges and demonstrated adequate dispersion for predictor screening. Full cohort characteristics are presented in Table 1.

### 3.2 Group comparison between FN and non-FN in the derivation cohort

Within the derivation cohort, patients were classified into FN and non-FN groups based on FN occurrence during the evaluable chemotherapy cycle (Table 2). Compared with non-FN patients, those who developed FN were older (60.4 ± 11.6 vs. 56.6 ± 12.0 years), had poorer functional status (ECOG ≥2: 26.9% vs. 15.3%), and more frequently had cardiovascular disease (25.0% vs. 16.0%) and diabetes mellitus (19.4% vs. 13.9%).

**TABLE 2:** Baseline characteristics of patients with and without febrile neutropenia in the derivation cohort.

Characteristic	No FN (n = 1,272)	FN (n = 216)	P value
Age, years, mean ± SD	56.6 ± 12.0	60.4 ± 11.6	<0.001
Female sex, n (%)	616 (48.4)	93 (43.1)	0.164
BMI, kg/m^2^, mean ± SD	23.8 ± 3.3	22.9 ± 3.6	0.002
ECOG 0–1, n (%)	1,078 (84.7)	158 (73.1)	<0.001
ECOG ≥2, n (%)	194 (15.3)	58 (26.9)	<0.001
Current/former smoker, n (%)	359 (28.2)	77 (35.6)	0.022
Cardiovascular disease, n (%)	204 (16.0)	54 (25.0)	0.001
Diabetes mellitus, n (%)	177 (13.9)	42 (19.4)	0.031
Chronic kidney disease, n (%)	71 (5.6)	19 (8.8)	0.061
Prior radiotherapy within 6 months, n (%)	302 (23.7)	60 (27.8)	0.198
Cancer type, n (%)	​	​	0.003
Lung	255 (20.0)	34 (15.7)	0.141
Breast	235 (18.5)	21 (9.7)	0.001
Colorectal	206 (16.2)	26 (12.0)	0.118
Gastric	151 (11.9)	28 (13.0)	0.642
Hematologic	152 (11.9)	48 (22.2)	<0.001
Gynecologic	132 (10.4)	18 (8.3)	0.349
Other	139 (10.9)	41 (19.0)	0.001
Cancer stage, n (%)	​	​	<0.001
Localized	327 (25.7)	33 (15.3)	0.001
Locally advanced	443 (34.8)	62 (28.7)	0.082
Metastatic	502 (39.5)	121 (56.0)	<0.001
Line of systemic therapy, n (%)	​	​	0.014
First line	870 (68.4)	126 (58.3)	0.004
Second line	292 (23.0)	60 (27.8)	0.123
Third line or later	110 (8.6)	30 (13.9)	0.009
Chemotherapy myelotoxicity risk, n (%)	​	​	<0.001
High	247 (19.4)	81 (37.5)	<0.001
Intermediate	650 (51.1)	95 (44.0)	0.054
Low	375 (29.5)	40 (18.5)	0.001
Cycle at enrollment, n (%)	​	​	<0.001
Cycle 1	394 (31.0)	98 (45.4)	<0.001
Cycles 2–3	542 (42.6)	77 (35.6)	0.061
Cycle ≥4	336 (26.4)	41 (19.0)	0.02
Prior febrile neutropenia history, n (%)	133 (10.5)	41 (19.0)	<0.001
Primary prophylactic G-CSF use, n (%)	322 (25.3)	27 (12.5)	<0.001
Antibacterial prophylaxis, n (%)	104 (8.2)	21 (9.7)	0.463
Inpatient chemotherapy administration, n (%)	457 (35.9)	92 (42.6)	0.053
Systemic corticosteroids during cycle, n (%)	144 (11.3)	41 (19.0)	0.001
Central venous catheter, n (%)	468 (36.8)	101 (46.8)	0.004
Mucositis grade ≥2, n (%)	172 (13.5)	50 (23.1)	<0.001
Nutrition risk (NRS 2002 ≥ 3 or PG-SGA B/C), n (%)	334 (26.3)	89 (41.2)	<0.001
Limited self-monitoring adherence*, n (%)	197 (15.5)	52 (24.1)	0.001
Baseline ANC, ×10^9^/L, median (IQR)	3.31 (2.67–4.10)	2.88 (2.29–3.67)	<0.001
Baseline WBC, ×10^9^/L, mean ± SD	5.94 ± 1.72	5.42 ± 1.83	<0.001
Hemoglobin, g/L, mean ± SD	119.4 ± 17.4	112.8 ± 18.5	<0.001
Platelets, ×10^9^/L, median (IQR)	228 (180–291)	201 (154–267)	<0.001
Albumin, g/L, mean ± SD	39.6 ± 4.6	37.5 ± 5.2	<0.001
eGFR, mL/min/1.73 m^2^, mean ± SD	93.5 ± 18.1	90.2 ± 19.6	0.018
Total bilirubin, µmol/L, median (IQR)	10.5 (7.8–14.4)	11.1 (8.1–15.6)	0.089
ALT, U/L, median (IQR)	22 (16–31)	24 (17–35)	0.011
C-reactive protein, mg/L, median (IQR)	4.2 (2.0–8.5)	7.6 (3.1–15.4)	<0.001
Neutrophil-to-lymphocyte ratio, median (IQR)	2.28 (1.60–3.25)	2.91 (2.01–4.18)	<0.001
Baseline temperature, °C, mean ± SD	36.70 ± 0.31	36.76 ± 0.33	0.006

“*” denotes limited self-monitoring adherence was defined as documented suboptimal adherence to temperature monitoring and/or delayed reporting of infection-related symptoms during the chemotherapy cycle.

Treatment- and disease-related factors also differed between groups. FN occurred more often in patients with metastatic disease (56.0% vs. 39.5%), in later lines of systemic therapy (third-line or later: 13.9% vs. 8.6%), and among those receiving high-myelotoxicity regimens (37.5% vs. 19.4%). Enrollment at cycle 1 (45.4% vs. 31.0%) and a prior FN history (19.0% vs. 10.5%) were more common in the FN group. In addition, primary prophylactic G-CSF use was less frequent among FN patients (12.5% vs. 25.3%), consistent with real-world heterogeneity in supportive care.

Nursing assessment variables showed clear separation. Central venous catheter use (46.8% vs. 36.8%), mucositis grade ≥2 (23.1% vs. 13.5%), nutritional risk (41.2% vs. 26.3%), and limited self-monitoring adherence (24.1% vs. 15.5%) were all more common in the FN group.

Baseline laboratory profiles were also less favorable among FN patients, including lower baseline ANC and WBC counts, lower hemoglobin and platelet levels, lower albumin levels, and higher inflammatory markers (CRP and neutrophil-to-lymphocyte ratio). These univariable comparisons were used to describe group-level differences and to inform subsequent predictor selection and multivariable modeling (Table 2).

### 3.3 Feature selection and candidate model development results

**FIGURE 3:**
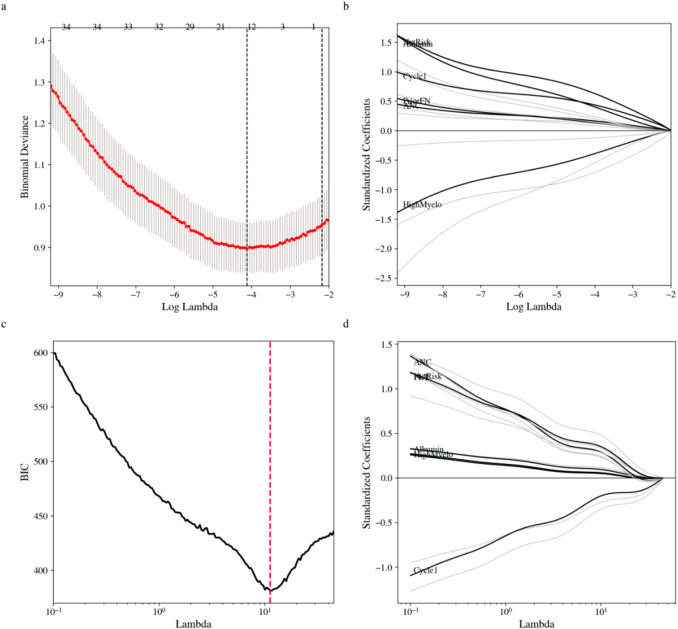
LASSO-based feature selection in the derivation cohort. **(a)** Cross-validation curve for binomial deviance across log lambda values. **(b)** Standardized coefficient paths across log lambda values. **(c)** BIC curve for lambda selection. **(d)** Standardized coefficient shrinkage paths across lambda values.

Predictor screening and candidate model construction were performed in the derivation cohort according to the prespecified predictor domains. To mitigate multicollinearity and reduce overfitting, we applied least absolute shrinkage and selection operator (LASSO) regression with K-fold cross-validation. The penalty parameter was evaluated using both the minimum cross-validated deviance and the 1-standard-error rule, and the 1-standard-error rule was selected to favor parsimony for bedside use. Regularization paths and the cross-validation curve are shown in Figure 3. As penalization increased, coefficients for weaker predictors shrank toward zero, yielding a parsimonious subset that captured regimen-related myelotoxic burden, baseline immune and nutritional reserve, and nursing-sensitive risk factors.

**FIGURE 4:**
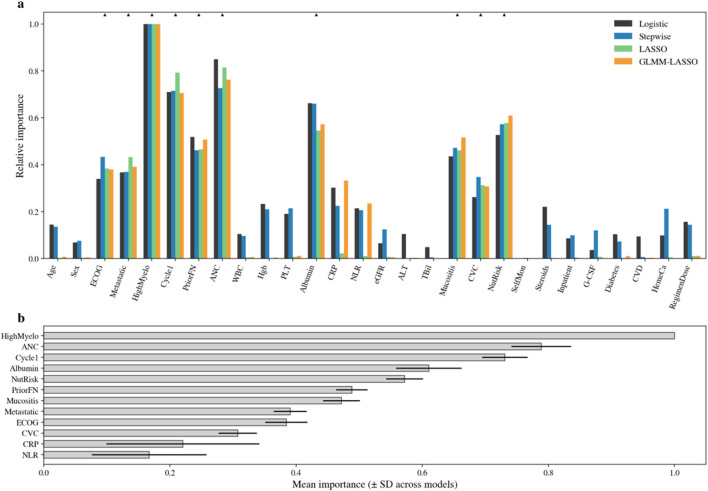
Relative importance of predictors across candidate models. **(a)** Relative importance of predictors across logistic, stepwise, LASSO, and GLMM-LASSO models. **(b)** Mean predictor importance ranking across candidate models.

We constructed several candidate models in parallel to examine the stability and interpretability of predictors across modeling strategies, including a standard multivariable logistic regression model, a stepwise logistic regression model, a LASSO-penalized model, and a generalized linear mixed-model LASSO (GLMM-LASSO) model. Predictor rankings across candidate models are summarized in Figure 4. Across approaches, regimen myelotoxicity risk, enrollment at cycle 1, prior FN history, baseline absolute neutrophil count (ANC), and serum albumin consistently emerged as influential predictors. Notably, nursing-sensitive factors, including nutritional risk, mucositis, and central venous catheter use, were also retained in penalized models, indicating independent contributions beyond conventional clinical and laboratory variables.

Regression directions were consistent across candidate models and aligned with clinical plausibility. Detailed coefficient estimates and corresponding odds ratios for key predictors in each candidate model are presented in Table 3. These comparisons supported the selection of a nursing-enhanced model that retained nurse-assessable variables while maintaining a simple and interpretable structure for subsequent visualization and validation.

**TABLE 3:** Predictor effects and model coefficients for febrile neutropenia risk across candidate models.

Predictor (coding/unit)	Logistic β	Logistic OR (95% CI)	Stepwise β	Stepwise OR (95% CI)	LASSO β	LASSO OR (95% CI)	GLMM-LASSO β	GLMM-LASSO OR (95% CI)
Age (per 10 years)	0.218	1.24 (1.12–1.38)	0.196	1.22 (1.10–1.36)	​	​	0.172	1.19 (1.06–1.33)
ECOG ≥2 (yes vs. no)	0.571	1.77 (1.29–2.43)	0.548	1.73 (1.26–2.39)	0.402	1.50 (1.10–2.05)	0.533	1.70 (1.22–2.37)
Metastatic disease (yes vs. no)	0.509	1.66 (1.23–2.25)	0.476	1.61 (1.19–2.18)	0.361	1.44 (1.07–1.95)	0.452	1.57 (1.15–2.15)
Hematologic malignancy (yes vs. solid tumors)	0.704	2.02 (1.43–2.85)	0.668	1.95 (1.37–2.77)	0.521	1.68 (1.20–2.35)	0.611	1.84 (1.30–2.62)
High-myelotoxicity regimen (high vs. non-high)	0.958	2.61 (1.95–3.49)	0.931	2.54 (1.89–3.42)	0.884	2.42 (1.85–3.18)	0.916	2.50 (1.88–3.33)
Cycle 1 at enrollment (yes vs. no)	0.693	2.00 (1.48–2.70)	0.661	1.94 (1.43–2.62)	0.641	1.90 (1.45–2.50)	0.654	1.92 (1.45–2.56)
Prior FN history (yes vs. no)	0.618	1.86 (1.29–2.68)	0.586	1.80 (1.24–2.61)	0.503	1.65 (1.18–2.29)	0.571	1.77 (1.25–2.49)
Baseline ANC (per 1.0 × 10^9^/L)	−0.346	0.71 (0.62–0.81)	−0.332	0.72 (0.63–0.82)	−0.318	0.73 (0.65–0.82)	−0.329	0.72 (0.63–0.81)
Albumin (per 5 g/L)	−0.291	0.75 (0.66–0.86)	−0.276	0.76 (0.67–0.87)	−0.244	0.78 (0.69–0.88)	−0.268	0.76 (0.67–0.86)
Nutrition risk (NRS 2002 ≥ 3 or PG-SGA B/C)	0.486	1.63 (1.20–2.20)	0.451	1.57 (1.15–2.13)	0.403	1.50 (1.12–2.01)	0.468	1.60 (1.18–2.17)
Mucositis grade ≥2 (yes vs. no)	0.552	1.74 (1.25–2.42)	0.523	1.69 (1.20–2.36)	0.441	1.55 (1.13–2.12)	0.538	1.71 (1.22–2.40)
Central venous catheter (yes vs. no)	0.316	1.37 (1.04–1.80)	​	​	​	​	0.284	1.33 (1.01–1.75)
CRP (per 10 mg/L)	0.147	1.16 (1.07–1.25)	0.139	1.15 (1.06–1.24)	​	​	0.128	1.14 (1.05–1.23)
Systemic corticosteroids during cycle (yes vs. no)	0.388	1.47 (1.06–2.04)	​	​	​	​	0.331	1.39 (1.01–1.92)
Primary prophylactic G-CSF use (yes vs. no)	−0.462	0.63 (0.44–0.89)	−0.438	0.65 (0.46–0.92)	−0.372	0.69 (0.51–0.93)	−0.401	0.67 (0.49–0.92)

### 3.4 Multivariable modeling and final predictor effects

Based on feature screening and candidate model comparison, we selected a nursing-enhanced multivariable logistic regression model as the final prediction model, prioritizing predictors that are routinely accessible in nursing practice and interpretable for clinical decision support. In the final model, high chemotherapy myelotoxicity risk, enrollment at cycle 1, and prior FN history were independently associated with increased FN risk, whereas higher baseline ANC and higher serum albumin levels were associated with lower risk, consistent with preserved immune and nutritional reserve.

Several nursing-sensitive predictors remained significant after adjustment for conventional clinical and laboratory variables. Nutritional risk and mucositis grade ≥2 were independently associated with higher FN risk, underscoring the relevance of impaired nutritional reserve and mucosal barrier injury. Central venous catheter use was also associated with increased risk, consistent with device-related infection pathways. Low self-monitoring adherence and higher baseline inflammatory burden, reflected by C-reactive protein (CRP), further increased FN risk, indicating actionable targets for intensified education, early warning, and symptom monitoring. Primary prophylactic G-CSF use was associated with lower FN risk, and ECOG performance status ≥2 remained an independent risk factor. Regression coefficients, adjusted odds ratios, 95% confidence intervals, and P values are summarized in Table 4.

**TABLE 4:** Multivariable logistic regression of nursing-relevant predictors for chemotherapy-induced febrile neutropenia.

Predictor (coding/unit)	β (SE)	Adjusted OR	95% CI	P value
High-myelotoxicity regimen (high vs. non-high)	0.905 (0.148)	2.47	1.85–3.30	<0.001
Cycle 1 (yes vs. no)	0.636 (0.131)	1.89	1.46–2.44	<0.001
Prior FN history (yes vs. no)	0.562 (0.165)	1.75	1.27–2.42	<0.001
Baseline ANC (per 1.0 × 10^9^/L)	−0.318 (0.053)	0.73	0.66–0.81	<0.001
Serum albumin (per 5 g/L)	−0.256 (0.061)	0.77	0.69–0.87	<0.001
Nutrition risk (NRS 2002 ≥ 3 or PG-SGA B/C)	0.438 (0.134)	1.55	1.19–2.02	0.001
Mucositis grade ≥2 (yes vs. no)	0.514 (0.152)	1.67	1.24–2.25	<0.001
Central venous catheter (yes vs. no)	0.287 (0.121)	1.33	1.05–1.69	0.018
Self-monitoring adherence low (yes vs. no)	0.402 (0.145)	1.49	1.13–1.99	0.006
CRP (per 10 mg/L)	0.118 (0.041)	1.13	1.04–1.22	0.004
Primary prophylactic G-CSF use (yes vs. no)	−0.388 (0.162)	0.68	0.49–0.93	0.017
ECOG ≥2 (yes vs. no)	0.371 (0.155)	1.45	1.07–1.96	0.017

### 3.5 Model performance in derivation and validation cohorts

**FIGURE 5:**
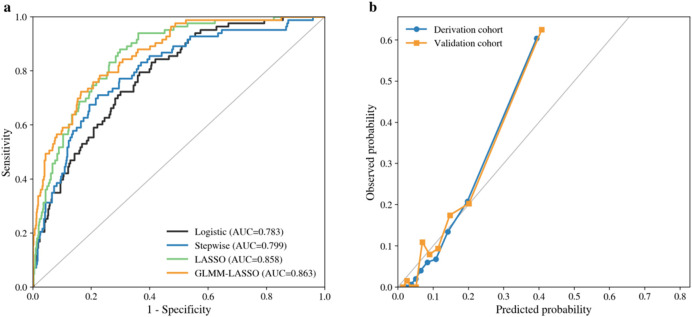
Discrimination and calibration performance of the final model. **(a)** ROC curves for the derivation and validation cohorts. **(b)** Calibration plot comparing predicted and observed FN probabilities in the derivation and validation cohorts.

The final nursing-oriented model demonstrated good discrimination and satisfactory calibration in both the derivation and validation cohorts (Figure 5). The AUC was 0.881 (95% CI, 0.854–0.907) in the derivation cohort and 0.861 (95% CI, 0.823–0.899) in the validation cohort, indicating stable discrimination with a modest decrease in performance on held-out data.

The FN event rate was comparable between cohorts (derivation: 216/1,488 [14.5%]; validation: 92/637 [14.4%]; Table 5), supporting the prespecified split for internal evaluation.

**TABLE 5:** Predictive performance of the febrile neutropenia risk model in the derivation and validation cohorts.

Metric	Derivation n=1,488	Validation n=637
FN events, n (%)	216 (14.5)	92 (14.4)
AUC (95% CI)	0.881 (0.854–0.907)	0.861 (0.823–0.899)
Brier score	0.086	0.090
Calibration intercept	−0.02	0.04
Calibration slope	0.97	0.93
Sensitivity	0.780	0.743
Specificity	0.750	0.730
PPV	0.327	0.316
NPV	0.953	0.944
Accuracy	0.754	0.732

Calibration assessment showed close agreement between predicted and observed FN risk across deciles of predicted probability in both cohorts (Figure 5). Calibration intercepts were near 0, and slopes were near 1 (derivation: −0.02 and 0.97; validation: 0.04 and 0.93), suggesting minimal systematic miscalibration. Brier scores were low (0.086 in derivation and 0.090 in validation), supporting good overall accuracy of predicted probabilities.

At the prespecified operating threshold used for classification, the model achieved a sensitivity of 0.780 and specificity of 0.750 in the derivation cohort, and 0.743 and 0.730 in the validation cohort. Negative predictive value remained high (derivation: 0.953; validation: 0.944), supporting safe identification of a larger low-risk group for routine follow-up while prioritizing high-risk patients for intensified monitoring (Table 5).

### 3.6 Nomogram development and calibration of the final model

**FIGURE 6:**
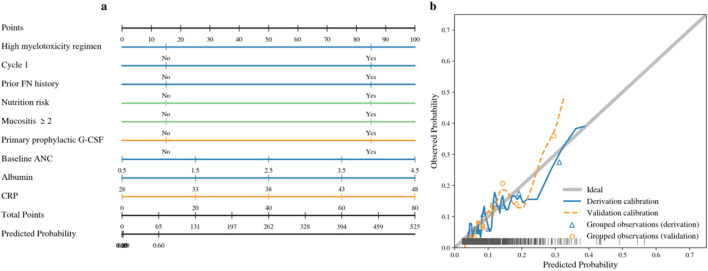
Nomogram derived from the final nursing-oriented model for individualized prediction of chemotherapy-induced febrile neutropenia **(a)**, with calibration plot **(b)**.

To facilitate bedside implementation, the final multivariable model was translated into a nomogram for individualized FN risk estimation using routinely available clinical, laboratory, and nursing assessment inputs. The nomogram integrates regimen myelotoxicity risk, cycle at enrollment, prior FN history, prophylactic G-CSF use, baseline ANC, serum albumin, CRP, and nursing-sensitive factors (nutritional risk and mucositis). Each predictor level is assigned a point value proportional to its regression coefficient, and the total points correspond to an estimated probability of FN during the evaluable chemotherapy cycle (Figure 6).

Point assignments for each predictor level are summarized in Table 6, enabling rapid bedside scoring. During routine assessment, nurses can determine regimen risk, cycle status, prior FN history, nutritional and mucosal status, prophylactic G-CSF use, and key baseline laboratory values, sum the corresponding points, and obtain an individualized predicted FN risk. This provides an operational basis for standardized risk stratification and subsequent tailored nursing interventions at the point of care (Chen et al., 2025).

**TABLE 6:** Final predictors included in the nursing-oriented model and corresponding point allocations in the nomogram.

Predictor	Level/value (coding)	Points
Regimen myelotoxicity	Low/moderate	0
​	High	64
Cycle	≥2	0
​	Cycle 1	46
Prior febrile neutropenia history	No	0
​	Yes	41
Nutrition risk (nursing assessment)	No	0
​	Yes	34
Mucositis (nursing assessment)	Grade 0–1	0
​	Grade ≥2	44
Primary prophylaxis (G-CSF)	Yes	0
​	No	31
Baseline ANC (×10^9^/L)	4.5	0
​	3.5	8
​	3	16
​	2.5	26
​	2	39
​	1.5	54
​	1	71
​	0.5	87
Albumin (g/L)	48	0
​	44	9
​	40	19
​	36	31
​	32	45
​	28	58
CRP (mg/L)	0	0
​	10	9
​	20	17
​	30	26
​	40	34
​	60	49
​	80	63

**FIGURE 7:**
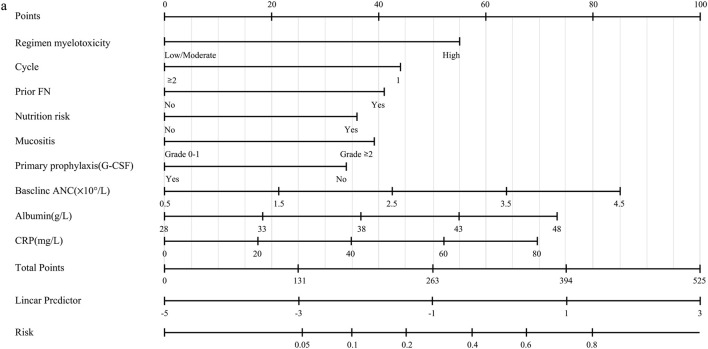
Nursing-based risk chart derived from the nomogram for bedside stratification of febrile neutropenia risk during chemotherapy.

To further support workflow integration, we derived a simplified nursing-specific risk chart from the same model (Figure 7). The chart links common combinations of key predictors to approximate risk ranges and is intended as a quick reference for decisions regarding monitoring intensity, education priorities, and escalation triggers. The calibration of nomogram-based predictions remained consistent with the cohort-level calibration findings reported above.

### 3.7 Clinical validation, decision utility, and risk-stratification outputs

**FIGURE 8:**
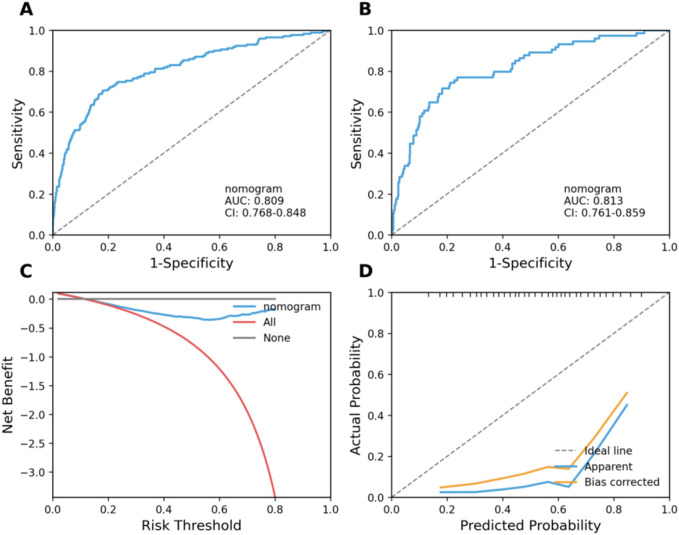
Clinical validation and clinical utility of the febrile neutropenia risk model. **(A)** ROC curve in the validation cohort. **(B)** Calibration plot in the validation cohort. **(C)** Decision curve analysis showing net benefit across a range of threshold probabilities for febrile neutropenia. **(D)** Distribution of patients across low-, intermediate-, and high-risk strata based on predicted probabilities.

In the validation cohort, the final nursing-oriented FN risk model maintained stable discrimination and calibration. The ROC curve remained well above the reference diagonal, indicating reliable separation between patients who developed FN and those who did not (Figure 8, Panel A). Calibration assessment showed close agreement between predicted probabilities and observed FN incidence across the risk spectrum, without evidence of systematic over- or underestimation (Figure 8, Panel B).

Clinical usefulness was examined using decision curve analysis (DCA). Across a clinically plausible range of FN threshold probabilities, the model yielded higher net benefit than default strategies of managing all patients as high risk (“treat-all”) or managing none (“treat-none”), supporting its value for triggering early nursing alerts and prioritizing surveillance resources under real-world constraints (Figure 8, Panel C). In this cohort, net benefit was consistently favorable across approximately 0.05–0.30 threshold probabilities, reflecting the range where escalation decisions are most sensitive to trade-offs between missed FN events and unnecessary intensive monitoring.

To translate predicted probabilities into bedside decision support, we categorized patients into three prespecified risk strata: low risk (<0.10), intermediate risk (0.10–0.25), and high risk (>0.25). These cutoffs were selected to (i) align with the threshold probability region where DCA indicated meaningful net benefit, and (ii) yield operationally distinct groups with separable observed FN incidence and downstream consequences relevant to nursing workload and escalation pathways. The resulting distribution placed 69.6% of patients in the low-risk group, 24.5% in the intermediate-risk group, and 5.9% in the high-risk group (Table 7; Figure 8, Panel D).

**TABLE 7:** Risk stratification by predicted probability and associated clinical outcomes of febrile neutropenia.

Item	Low risk	Intermediate risk	High risk	Total
Predicted probability range	<0.10	0.10–0.25	>0.25	​
Median predicted probability (IQR)	0.06 (0.04–0.08)	0.16 (0.12–0.21)	0.34 (0.29–0.46)	​
N (%)	1,480 (69.6)	520 (24.5)	125 (5.9)	2,125 (100)
Observed FN, n (%)	110 (7.4)	140 (26.9)	58 (46.4)	308 (14.5)
Odds ratio for FN vs. low (95% CI)	Reference	4.59 (3.50–6.02)	10.78 (7.10–16.37)	​
Hospital admission, n (%)	138 (9.3)	146 (28.1)	62 (49.6)	346 (16.3)
ICU admission, n (%)	6 (0.4)	10 (1.9)	7 (5.6)	23 (1.1)
Blood culture obtained, n (%)	210 (14.2)	148 (28.5)	53 (42.4)	411 (19.3)
Documented BSI, n (%)	18 (1.2)	22 (4.2)	14 (11.2)	54 (2.5)
Length of stay, days (median, IQR)	3.2 (2.0–5.1)	5.1 (3.4–7.8)	7.6 (5.2–10.9)	​
Chemo delay or dose reduction, n (%)	164 (11.1)	110 (21.2)	43 (34.4)	317 (14.9)
30-day all-cause mortality, n (%)	9 (0.6)	6 (1.2)	5 (4.0)	20 (0.9)

Risk strata showed a clear gradient in FN incidence. Observed FN rates increased from 7.4% in the low-risk group to 26.9% in the intermediate-risk group and 46.4% in the high-risk group, yielding an overall FN incidence of 14.5% (308/2,125). Relative to the low-risk group, the odds ratio for FN was 4.59 (95% CI, 3.50–6.02) in the intermediate-risk group and 10.78 (95% CI, 7.10–16.37) in the high-risk group (Table 7).

Importantly, the same stepwise pattern extended to clinically consequential outcomes that directly inform nursing workload and escalation pathways. Hospital admission increased from 9.3% to 28.1% and 49.6% across strata, and ICU admission increased from 0.4% to 1.9% and 5.6%, respectively. Measures reflecting infection work-up and severity also rose stepwise, including blood culture acquisition (14.2%, 28.5%, and 42.4%) and documented bloodstream infection (1.2%, 4.2%, and 11.2%). The median length of stay increased from 3.2 days in the low-risk group to 5.1 days in the intermediate-risk group to 7.6 days in the high-risk group. Treatment disruption similarly followed the risk gradient, with chemotherapy delays and dose reductions rising from 11.1% in the low-risk group to 21.2% in the intermediate-risk group and 34.4% in the high-risk group. Thirty-day all-cause mortality was highest in the high-risk group (0.6%, 1.2%, and 4.0%, respectively).

Collectively, these findings indicate that the model provides not only statistically robust FN risk prediction but also clinically meaningful stratification outputs that track with downstream consequences. This supports its intended role as a nursing-oriented decision-support tool to differentiate monitoring intensity, reinforce self-monitoring and education, and standardize escalation triggers according to predicted risk level.

### 3.8 Risk-stratified nursing pathways and practical implementation outputs

To operationalize the model outputs for oncology nursing workflows, we translated the three-tier risk strata into a structured set of risk-stratified nursing pathways. The intent is to convert a predicted probability into standardized and auditable actions spanning patient education, monitoring intensity, escalation triggers, and coordination with the oncology team. This “prediction-to-action” mapping is designed to be implementable using routine nursing documentation items (e.g., mucositis grading, nutritional screening, and self-monitoring adherence) and commonly available baseline laboratory results, thereby preserving feasibility in real-world settings.

At the point of care (typically at cycle initiation or within the early-cycle window), nurses can generate an individualized FN risk estimate using the nomogram (Figure 6) or the simplified risk chart (Figure 7). Patients are then assigned to low-, intermediate-, or high-risk strata (Table 7). Each stratum corresponds to a predefined bundle of nursing actions with increasing intensity. In brief, low-risk patients receive standard infection-prevention education and routine follow-up; intermediate-risk patients receive reinforced education, structured self-monitoring plans, and scheduled follow-up contacts; and high-risk patients receive intensified surveillance with explicit escalation thresholds, closer post-discharge follow-up, and proactive coordination for supportive care planning.

To increase transparency and reproducibility, Table 8 summarizes the recommended nursing actions for each stratum. The bundles emphasize modifiable and nursing-sensitive targets identified by the model, including mucosal barrier protection, nutritional support, catheter care, and adherence to temperature monitoring and early symptom reporting. Escalation triggers are framed as “if–then” rules to reduce variability across nurses and shifts and to support timely activation of physician review and supportive measures. This structured mapping provides a practical template for implementing precision preventive nursing informed by the FN risk model.

**TABLE 8:** Risk-stratified nursing pathway recommendations linked to predicted FN risk.

Domain	Low risk (<0.10)	Intermediate risk (0.10–0.25)	High risk (>0.25)
Core education	Standard FN prevention education; reinforce hand hygiene and infection-avoidance behaviors	Reinforced education with teach-back; document understanding and barriers	Intensive education + caregiver involvement; confirm teach-back and provide written action plan
Temperature monitoring	Daily self-check (or per routine institutional protocol)	Twice-daily self-check for first 10–14 days of cycle; document adherence plan	Twice-daily self-check + symptom diary; proactive nurse follow-up during expected nadir window
Follow-up intensity	Routine follow-up schedule	Scheduled follow-up contact during mid-cycle (e.g., day 7–10) to verify symptoms/adherence	Early and repeated contacts during the nadir window; consider a dedicated hotline verification and a rapid access pathway
Mucositis and barrier care	Routine oral care guidance	Standardized oral care protocol; early management for grade 1 changes	Intensified barrier protection; prioritize prompt assessment for grade ≥2 mucositis and coordinate escalation
Nutritional support	Routine screening per workflow	Targeted nutrition counseling for at-risk patients; follow-up on intake	Prioritize nutrition consultation; frequent reassessment for nutritional risk and hydration status
Catheter-related care	Routine catheter care, if present	Reinforce catheter care education; inspect/document signs of infection	Enhanced catheter surveillance; low threshold for evaluation if any local/systemic signs
Escalation triggers	Fever meeting FN criteria or concerning symptoms prompt immediate contact	Lower threshold for contact (e.g., persistent malaise, chills, and poor intake) + fever criteria	Explicit “urgent evaluation” triggers (fever criteria, rigors, hypotension symptoms, and rapid deterioration) with an immediate escalation pathway
Coordination with the oncology team	Standard coordination as needed	Communicate intermediate-risk status at cycle start; align monitoring plan	Flag high-risk status at cycle start; proactive coordination for supportive care planning and rapid response pathway
Documentation outputs	Record risk stratum and education provided	Record stratum + follow-up plan + adherence assessment	Record stratum + escalation plan + nadir-window follow-up schedule

## 4 Discussion

This study focused on early identification of febrile neutropenia (FN) during chemotherapy and on supporting nursing decision-making through a nursing-oriented prediction model (Zhu et al., 2024; Gascón et al., 2024). Based on 2,125 evaluable chemotherapy cycles, a multivariable model integrating treatment intensity, clinical history, key laboratory indicators, and nurse-assessed risk factors was developed and internally validated using a prespecified split-sample approach. The model showed stable discrimination, good calibration, and consistent net clinical benefit across clinically plausible thresholds (Shah et al., 2025). These findings suggest that the tool is not only statistically robust but also practically relevant for risk-stratified nursing management in a population where FN risk is heterogeneous and cannot be adequately captured by chemotherapy regimen risk alone (Leon Rapoport et al., 2023).

Compared with previously reported FN prediction tools, this work addresses limitations highlighted in recent systematic reviews (Liu et al., 2025). Many earlier models were developed in smaller cohorts, used heterogeneous endpoint definitions, or lacked validation beyond the development sample, which limits transportability and clinical uptake. In addition, widely used instruments such as the Multinational Association of Supportive Care in Cancer (MASCC) risk index and other post-FN complication scores are primarily designed to stratify risk after FN has occurred and are often used to guide site-of-care decisions rather than to prevent FN during chemotherapy cycles. In contrast, the present model targets pre- or peri-cycle prediction of FN itself, was evaluated in a prespecified validation cohort, and integrates discrimination, calibration, and decision curve analysis (DCA) within a unified performance framework (Yoshinami et al., 2025). These features help move the model from a purely “paper tool” toward a candidate workflow-ready tool that can support routine nursing practice (Jean et al., 2025; Peng et al., 2025).

A key contribution of this study lies in its nursing-oriented predictor framework and its incremental value relative to clinician-focused models. We intentionally prioritized predictors that are observable, interpretable, and actionable within nursing workflows (Fowler et al., 2025). While regimen myelotoxicity risk, cycle 1 exposure, prior FN history, and baseline physiological reserve markers (e.g., ANC and albumin) are well-established FN predictors (Deng et al., 2025), our final model also retained nurse-sensitive domains, including nutritional risk, mucositis, central venous catheter exposure, and self-monitoring adherence, after adjustment for treatment intensity and baseline laboratory values. This demonstrates that nursing-assessable factors provide additional risk information that can be mapped directly to modifiable care targets and preventive nursing actions, rather than duplicating clinician-only predictors (Wondm et al., 2025).

The mechanisms underpinning these associations are biologically and clinically plausible. Higher regimen myelotoxicity, first-cycle chemotherapy, metastatic disease, and prior FN history reflect greater exposure to bone marrow suppression and infection stress (Aslam et al., 2023). Lower baseline ANC and albumin signal reduced immune and nutritional reserve, potentially limiting tolerance to cytotoxic injury and response to infection. From a nursing perspective, nutritional risk and grade ≥2 mucositis reflect compromised mucosal integrity and impaired barrier function, while central venous catheters represent device-related portals of entry that may increase infection susceptibility. Limited self-monitoring adherence plausibly delays recognition and early reporting of fever, narrowing the window for timely evaluation and escalation. Elevated C-reactive protein may capture a pro-inflammatory baseline state associated with infectious vulnerability. Taken together, these findings support a multifactorial FN risk pathway shaped by treatment intensity, host reserve, barrier integrity, and health behaviors, dimensions that are directly relevant to oncology nursing assessment and intervention (Pal et al., 2023).

In terms of clinical application, guideline-based FN prevention emphasizes risk assessment to inform primary prophylaxis (e.g., G-CSF), infection surveillance, triage thresholds for urgent evaluation, and follow-up scheduling. The DCA results in this study suggest that model-guided strategies can provide greater net benefit than blanket strategies of treating all patients as high risk or none as high risk within commonly considered threshold ranges, supporting selective intensification for those at truly elevated risk while avoiding unnecessary escalation in low-risk patients.

Risk stratification further illustrates how prediction outputs can be translated into operational nursing pathways. Low-risk patients exhibited relatively low FN incidence and fewer downstream adverse outcomes, suggesting that routine monitoring, standardized infection-prevention education, and reinforcement of home temperature recording may be sufficient. In contrast, intermediate- and high-risk patients showed substantially higher rates of FN and adverse outcomes, supporting intensified symptom/temperature surveillance, repeated assessment of oral mucosa and catheter care risks, systematic nutritional reassessment, and lower thresholds for urgent medical review when early-warning signs appear. This enables a closed-loop “risk assessment–stratified intervention–outcome tracking” approach that aligns model outputs with feasible nursing actions and resource allocation.

Importantly, this model is positioned for early-cycle risk stratification. Because we used the first evaluable chemotherapy cycle per patient, the model is optimized to support initial preventive nursing planning and early-cycle resource allocation, but it may underestimate cumulative toxicity and evolving FN risk in later cycles. This design choice improves independence of observations and standardizes predictor ascertainment relative to outcome assessment, but it should be considered when interpreting risk estimates across the full chemotherapy course (Pal et al., 2023).

Beyond FN occurrence, downstream complications are often driven by bloodstream infection, resistant pathogens, and severity trajectories. Data-driven approaches integrating microbiological and clinical phenotypes have begun to identify subgroups at particularly high risk of severe infectious complications, which may inform escalation planning and resource prioritization. The present model, although focused on FN occurrence, may function as an upstream layer that supports earlier surveillance and education before events arise, and it could be paired in the future with downstream tools that refine risk among patients who develop FN to enable stepwise decision support across the FN care pathway (Al-Aamri et al., 2025).

This study has several limitations. First, it is a retrospective single-center study, and nursing assessment variables may reflect local documentation practices and workflow structures, potentially affecting transportability. Second, despite prespecified missing-data handling and internal validation, residual confounding cannot be excluded. Third, the model is largely static, relying on baseline or early-cycle information rather than time-updated trajectories of symptoms and laboratory measures.

Future work should therefore include multicenter prospective validation in diverse settings, with recalibration or updating as needed (Borgeaud et al., 2024). Incorporating dynamic data streams, such as standardized nursing assessment scales, patient-reported symptom trajectories, and remote temperature monitoring, may enable time-updated prediction tools that better reflect within-cycle risk evolution. In addition, advances in precision analytics and detection technologies may inform future model optimization and clinical translation. For example, deep supervised learning approaches have been used to support early detection and improved outcomes in oncology contexts, illustrating a broader “precision analytics → outcome optimization” logic that can inspire future nursing decision-support development. Similarly, emerging electrochemiluminescence and nanomaterial-based sensing strategies highlight potential avenues to improve the detection precision of biomarkers relevant to infection risk monitoring. Finally, biomaterial reviews describing antimicrobial, anti-inflammatory, and immunomodulatory activities of iron oxide nanoparticles suggest longer-term translational directions for adjunctive prevention research in immunocompromised populations, which could eventually interface with personalized preventive care pathways when supported by rigorous safety evaluation and clinical validation.

## 5 Conclusion

In summary, this study developed and internally validated a nursing-oriented risk prediction model for febrile neutropenia in patients undergoing chemotherapy. By combining chemotherapy regimen intensity and treatment history with key laboratory indicators and nurse-assessed risk factors, the model achieved high discrimination, good calibration, and meaningful net clinical benefit in both derivation and validation cohorts (Leon Rapoport et al., 2023). Predicted risk strata showed graded associations not only with FN incidence but also with hospitalization, ICU admission, bloodstream infection, length of stay, chemotherapy delay or dose reduction, and short-term mortality.

These findings support the model’s potential as an early-warning tool for chemotherapy cycles, enabling individualized risk estimation and more precise allocation of nursing resources within routine workflows. By retaining nurse-sensitive predictors, the model also identifies actionable targets for patient education, barrier and catheter care, nutritional support, and escalation planning. Further multicenter prospective validation and implementation studies are warranted to confirm generalizability, to refine dynamic risk assessment across cycles, and to determine whether model-guided nursing bundles reduce FN incidence and improve patient-centered outcomes in real-world practice.

## Data Availability

The raw data supporting the conclusions of this article will be made available by the authors, without undue reservation.
