# Correction: Development and clinical validation of a nursing risk prediction model for chemotherapy-induced febrile neutropenia in patients with cancer

**DOI:** 10.3389/fcell.2026.1896927

**Published:** 2026-07-03

**Authors:** Fang Xie, Dongmei Zheng, Na Chen, Jie Zhang, Nan Wang, Lihuai Wang, Qin Yi

**Affiliations:** 1 Cancer Center, The First Hospital of Hunan University of Chinese Medicine, Changsha, Hunan, China; 2 Hematological Oncology Department, The First Hospital of Hunan University of Chinese Medicine, Changsha, Hunan, China; 3 Hunan University of Chinese Medicine, Changsha, Hunan, China

**Keywords:** chemotherapy, clinical prediction model, febrile neutropenia, oncology nursing, risk stratification

The order of authors in the author list of the published paper was erroneously given as:

Fang Xie¹, Dongmei Zheng², Na Chen¹, Jie Zhang³, Nan Wang¹, Qin Yi²†*, Lihuai Wang¹†*

The correct author list reads:

Fang Xie^1^, Dongmei Zheng^2^, Na Chen^1^, Jie Zhang^3^, Nan Wang^1^, Lihuai Wang^1^†*, Qin Yi^2^†*

The author contributions statement was erroneously given as FX: Conceptualization, Data curation, Formal analysis, Investigation, Methodology, Writing – original draft. DZ: Investigation, Methodology, Writing – original draft. NC: Project administration, Supervision, Validation, Writing – review and editing. JZ: Project administration, Resources, Software, Writing – review and editing. NW: Data curation, Formal analysis, Funding acquisition, Writing – review and editing. QY: Formal analysis, Funding acquisition, Investigation, Supervision, Writing – review and editing. LW: Methodology, Project administration, Resources, Writing – review and editing.

The correct author contributions statement is FX: Conceptualization, Data curation, Formal analysis, Investigation, Methodology, Writing – original draft. DZ: Investigation, Methodology, Writing – original draft. NC: Project administration, Supervision, Validation, Writing – review and editing. JZ: Project administration, Resources, Software, Writing – review and editing. NW: Data curation, Formal analysis, Funding acquisition, Writing – review and editing. LW: Formal analysis, Funding acquisition, Investigation, Supervision, Writing – review and editing. QY: Methodology, Project administration, Resources, Writing – review and editing.

The original article has been updated.

